# *Ocimum sanctum* Linn. stimulate the expression of choline acetyltransferase on the human cerebral microvascular endothelial cells

**DOI:** 10.14202/vetworld.2016.1348-1354

**Published:** 2016-12-03

**Authors:** Dwi Liliek Kusindarta, Hevi Wihadmadyatami, Aris Haryanto

**Affiliations:** 1 Department of Anatomy, Faculty of Veterinary Medicine, Universitas Gadjah Mada, Yogyakarta, Indonesia; 2 Department of Biochemistry, Faculty of Veterinary Medicine, Universitas Gadjah Mada, Yogyakarta, Indonesia

**Keywords:** choline acetyltransferase, human cerebral microvascular endothelial cells, *Ocimum sanctum* Linn

## Abstract

**Aim:**

This research was conducted to identify the expression of choline acetyltransferase (ChAT) in human cerebral microvascular endothelial cells (HCMECs) and to clarify the capability of *Ocimum sanctum* Linn. ethanolic extract to stimulate the presence of ChAT in the aging HCMECs.

**Materials and Methods:**

In this study, we perform an *in vitro* analysis some in the presence of an ethanolic extract of *O. sanctum* Linn. as a stimulator for the ChAT expression. HCMECs are divided become two groups, the first is in low passage cells as a model of young aged and the second is in a high passage as a model of aging. Furthermore to analysis the expression of ChAT without and with extract treatments, immunocytochemistry and flow cytometry analysis were performed. In addition, ChAT sandwich enzyme-linked immunosorbent assay is developed to detect the increasing activity of the ChAT under normal, and aging HCMECs on the condition treated and untreated cells.

**Results:**

In our *in vitro* models using HCMECs, we found that ChAT is expressed throughout intracytoplasmic areas. On the status of aging, the ethanolic extract from *O. sanctum* Linn. is capable to stimulate and restore the expression of ChAT. The increasing of ChAT expression is in line with the increasing activity of this enzyme on the aging treated HCMECs.

**Conclusions:**

Our observation indicates that HCMECs is one of the noncholinergic cells which is produced ChAT. The administrated of *O. sanctum* Linn. ethanolic extract may stimulate and restore the expression of ChAT on the deteriorating cells of HCMECs, thus its may give nerve protection and help the production of acetylcholine.

## Introduction

The memory could be defined as an individual capability to record, retain, recall information and to use them in response to the environment [1]. During several years, there is a lot of problems were occur concerning memory impairment especially on the condition of aging known as dementia. Data from World Alzheimer Report proposed that in develop and developing countries increasing the number of life also give additional problems, tremendously increasing person suffering from neurodegenerative disease, e.g., Alzheimer disease and Parkinson are occurring and its already predicted in the year of 2040 will be reach become 135.5 million people [2]. Neurodegenerative diseases are specifically characterized by the presence of nonreversible disturbance mainly concerning on memory and cognitive impairment in relations to dementia. Dementia may give bad impact not only on the daily activities but also remarkable as a severe neurodegenerative clinical sign. Mainly developing of memory are based on neuronal plasticity or interaction between two or more neuron also known as long-term potentiation [3,4], which may release neurotransmitter. Acetylcholine (Ach) is ones of neurotransmitter which overstated play a pivotal role during learning and memory processes [5,6]. Choline acetyltransferase (ChAT) is an enzyme responsible for the Ach biosynthesis in the cytoplasm, thus the inhibition of ChAT production because of aging or pathological causes may impair the production of Ach and interferes memory as well as cognitive capability [7-10].

Until today treatment and prevention regarding dementia such as piracetam and anticholinesterases still have been yet giving satisfied results [11]. Therefore, there is needed to find a new solution on the medication, primarily to prevent dementia. This drug should be easy to find, not expensive and decrease or avoid the side effect of the chemical-based medicine.

*Ocimum sanctum* Linn. also known in Indonesia as kemangi is one of the potential herbs used as a medication. This herb belongs to the family Lamiaceae and easily found and grows throughout Indonesia. Have been reported, various parts from *O. sanctum* Linn. provide several advantages as an anti-inflammatory [12], antiallergic [13], antioxidant [14,15], radioprotective [16], and anticarcinogenic [17]; however until today, there is only restricted research explore how is the mechanism of *O. sanctum* Linn. act as neuroprotective [11,18]. In addition, recently have not been yet established, actually, how is the mechanism involving ChAT in noncholinergic neuronal nerve on the learning and memory in cellular level. Thus, this research is necessary to give new perspective in the importance of dementia medication.

This study was designed to analysis the ability of an ethanolic extract derived from the leaf of *O. sanctum* Linn. to stimulate the expression of enzyme ChAT on the human cerebral microvascular aging cells. In here, we postulated that increasing the expression of ChAT in noncholinergic cells also may involve to enhance and to restore memory and cognitive ability in aging individual.

## Materials and Methods

### Ethical approval

The use of all preclinical research material was approved by the Ethics Committee of Universitas Gadjah Mada, Yogyakarta, Indonesia.

### Preparation of extract

#### Crude extract of O. sanctum

The leafs of *O. sanctum* Linn. were purchased from CV. Merapi Herbal Farma, Yogyakarta, Indonesia. The analysis of the plant identities was done in Faculty of Biology, Universitas Gadjah Mada, Indonesia. The plants material was dried at the sunshine temperature (35-38°C) in outdoor conditions and powdered using commercial electric blender.

#### Ethanolic extract of O. sanctum

The powdered material of *O. sanctum* Linn. leafs (approximately about 100 g) exhaustively extracted with 90% ethanol in a Soxhlet’s apparatus until the solvent became apparent. The extract was concentrated and dried at 60-70°C. The extract percentage as the semi-solid extract was weight, place in tubes and store at 4°C until used.

### Monoclonal antibodies (MAbs)

Antibodies goat antiChAT (clone AB 144P) was purchased from Millipore, Temecula, CA, USA. MAbs goat antihuman platelet and endothelial cell adhesion molecule 1 (PECAM-1) (Clone SC 1506) was purchased from Santa Cruz, Heidelberg, Germany. MAb Gi5 against integrin αIIbβ3 was generously gift from Dr. Sentot Santoso, Ph.D. Giessen, Germany. Goat antimouse Alexa Fluor^®^ 488 and donkey antigoat Alexa Fluor^®^ 488 was derived from Thermo Fischer Scientific, Rockford, IL, USA.

### Maintenance of human cerebral microvascular endothelial cells (HCMECs)

HCMECs were purchased from Lonza, Bazel, Switzerlandand maintained in endothelial basal medium-2 (EBM-2; Lonza, Bazel, Switzerland). All experiments represent young ages were performed with low passages (primary, secondary or tertiary) postconfluent monolayers HCMECs, in addition high passage (15-20 passages) postconfluent monolayers HCMECs were used as old ages or aging conditioned [19,20]. In some experiments, high passage HCMECs were cultured with EBM-2 medium in the presence of 80 µg/ml ethanolic extract of *O. sanctum* Linn. for 2-3 weeks.

### Flow cytometry analysis

HCMECs were cultured for 1 week in EBM-2 medium, and the expression of ChAT was measured by flow cytometry (FACS Canto, Becton Dickinson, Heidelberg, Germany). Cells were incubated with 20 mL of MAb goat antihuman ChAT (Millipore, Temecula, CA, USA) for 30 min at 4°C, mouse immunoglobulin G (IgG) (Thermo Fischer Scientific, Rockford, IL, USA) was run as a control. After being washed with phosphate-buffered saline (PBS) buffer containing 0.5% bovine serum albumin (BSA), cells were labeled with fluorescein isothiocyanate (FITC)-conjugated anti-goat IgG antibody (dilution 1:100; Thermo Fischer Scientific, Rockford, IL, USA), washed, and fixed by CellFix™ (Becton Dickinson, Heidelberg, Germany).

### Immunocytochemistry

Aliquots of 300 µl HCMECs (10^5^ cells) were plated on 8-well µ-slides (IBIDI GmbH, Munich, Germany) for 24 h. Cells were fixed with 4% paraformaldehyde (Sigma-Aldrich, Munich, Germany), and stained with 300 µl goat anti-ChAT or mouse IgG (as isotype control, Santa Cruz, Heidelberg, Germany) and human PECAM-1 (CD 31, Santa Cruz, Heidelberg, Germany). Labeled cells were visualized using FITC-labeled rabbit-antimouse IgG (Dako) or FITC-labeled donkey antigoat IgG (Thermo Scientific) and PE-labeled donkey antigoat IgG (Dako). Cell nucleoli were stained using 4’,6-diamidino-2-phenylindole (Hoechst 33342; Thermo Scientific, Rockford, IL, USA) for 3 min at RT. Cells were visualized using confocal microscopy with ×40 magnification (Nikon Eclipse TE2000-E, Tokyo, Japan).

### ChAT enzyme-linked immunosorbent assay (ELISA)

In house sandwich, ELISA was developed to detect the activity of ChAT in the HCMECs. Subsequently 100 µl goat antihuman (5 mg) were coated overnight at 4°C. After being washed 3 times with PBS containing 0.2% (BSA; washing buffer), the wells were blocked with 100 µL of PBS supplemented with 2% BSA for 30 minutes at 4°C. Aliquots of 100 µL of cell lysates were added to the plate and incubated for 1 h at room temperature. Wells were washed 3 times and 100 µL of goat anti human ChAT was added for 60 min at 4°C. After washing 3 times, 100 µL of horseradish peroxidase-labeled donkey antigoat IgG (dilution 1:40,000; Sigma) was added for 1 h at 37°C. After washings, 100 µL of 3,3’,5,5’-tetramethyl benzidine substrate solution (Sigma) was added. The reaction was stopped after 20 min of incubation at room temperature with 50 µL of 1.0 mol/L HCl and was read at 450 nm on an ELISA reader. To correct for nonspecific binding, backgrounds (negative control) were run for cell lysate sample in parallel without antigen under identical conditions. Specific antibody binding was calculated as reaction rate by subtracting the individual background signal from the total binding. Cutoff values were determined as the mean signal of negative control (n=10) plus three standard deviations.

### Statistical analysis

Statistical comparisons were made using an unpaired, two-tailed Student’s t-test as appropriate. A p<0.05 was assumed to represent statistical significance. Statistic was performed using Graph Pad Prism 6 (La Jolla, CA, USA).

## Results

### ChAT was expressed intracytoplasmic on the HCMECs

**Figure-1:**
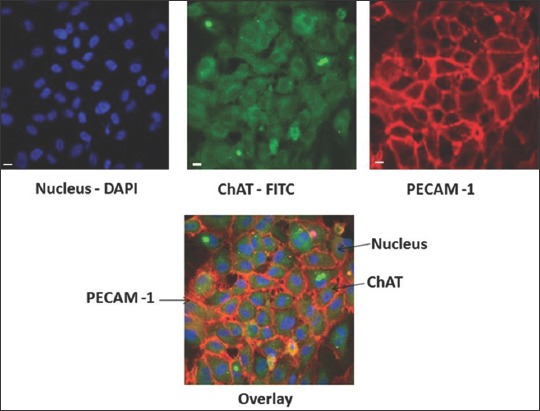
Identification of choline acetyltransferase (ChAT) expression on human cerebral microvascular endothelial cells (HCMECs) by immunocytochemistry. HCMECs were fixed and continued with incubation using primer antibody goat anti ChAT (Clone AB144P) and goat anti human platelet and endothelial cell adhesion molecule 1 (PECAM-1) (Clone SC 1506). After overnight incubation, cells were washed and labeled by secondary antibody (Alexa fluor FITC labeled donkey anti goat or PE donkey anti goat immunoglobulin G), nucleus were stained with 4’,6-diamidino-2-phenylindole. Blue=Nucleus; Green=ChAT; Red=PECAM-1.

The analysis by immunofluorescence on the HCMECs in the presence of PECAM-1 in the surrounding of cells membrane and or placed on the cells membrane junction [19,20] describe that ChAT was hampered on the intracytoplasmic area of the cells (Figure-1). These results indicating that ChAT was expressed on the HCMECs.

### Ethanolic extract O. sanctum Linn. could stimulate the expression of ChAT on the aging endothelial cells

**Figure-2:**
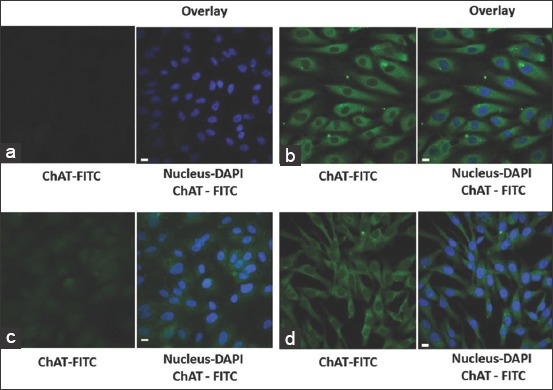
Choline acetyltransferase (ChAT) expression on the human cerebral microvascular endothelial cells (HCMECs) mimics Aging conditioned after and before treatment with *Ocimum sanctum* Linn. ethanolic extract. HCMECs were fixed and continued with incubation by using primer antibody goat anti ChAT (Clone AB144P). Mouse anti human integrin αIIbβ3 was run as a negative control. After overnight incubation, cells were washed and labeled by secondary antibody Alexa fluor fluorescein isothiocyanate (FITC) labeled donkey anti goat or Alexa fluor FITC labeled goat anti mouse, nucleus were stained with 4’,6-diamidino-2-phenylindole. (a) Negative control, (b) positive control ChAT expression, (c) untreated cells (d) treated cells. Blue=Nucleus; Green=ChAT.

After administrated of an ethanolic extract derived from the leafs of *O. sanctum* Linn., HCMECs were stained to visualize the ChAT expression. Integrin αIIbβ3 which is not expressed on the HCMECs were run as a negative control (Figure-2a) [21], whereas young age model cells act as the positive control for ChAT expression (Figure-2b). On the untreated aging cells, we can describe in here that there is almost not detected ChAT expression (Figure-2c), in contrast on the aging model cells with treatment, observed that ethanolic extract *O. sanctum* Linn. capable to stimulate and restore the expression of ChAT (Figure-2d), this results also showed in semiquantitative ChAT expression on Table-1.

**Table-1:** The semiquantitative number of ChAT expressions.

Cell conditions	HCMECs		

	Young	Old	Old and *O. sanctum* Linn. extract
ChAT expressions	+++	+	+++

+++=Numerous, -=Not detected, +=A few, ChAT=Choline acetyltransferase, HCMECs=Human cerebral microvascular endothelial cells, *O. sanctum*=*Ocimum sanctum*

**Figure-3:**
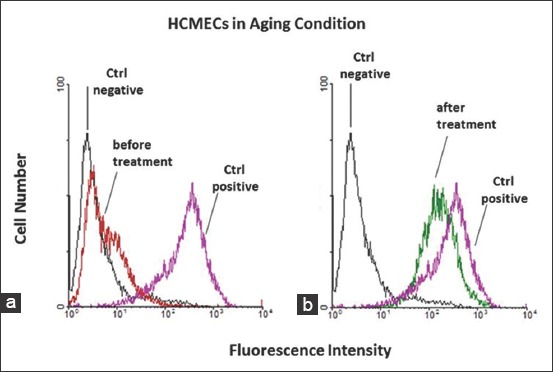
(a,b) Flow cytometry analysis of choline acetyltransferase (ChAT) expression on the human cerebral microvascular endothelial cells (HCMECs) mimics aging conditioned. HCMECs after and before treatment with ethanolic extract *Ocimum sanctum* Linn. in high passage were incubated with primer antibody goat anti ChAT (Clone AB144P). Isotype mouse immunoglobulin G (IgG) was run as a negative control, whereas low passage HCMECs was run as positive control ChAT expressions. After being washed, cells were labeled by secondary antibody (Alexa fluor labeled donkey anti goat or goat anti mouse IgG). Labeled cells were analyzed by flow cytometry. Black lines=Negative control; purple line=Positive control; red lines=HCMECs without treatment; green lines=HCMECs with treatment.

To confirm the expression of ChAT on the cells, flow cytometry analysis (FACS) was performed, and as expected in the FACS result, after treatment on the aging endothelial cells in comparison to the control, the expression of ChAT is increasing (mean fluorescent intensity [MFI]; 158.05 vs. 3.04) (Figure-3b) and almost reach the same expression of young age type cells (MFI; 228.83 vs. 3.04) (Figure-3b). Moreover, there is less ChAT expression on the untreated aging cells (MFI; 5.99 vs. 3.04) (Figure-3a).

### Ethanolic extract O. sanctum Linn. increases the activity of ChAT on the aging endothelial cells

**Figure-4:**
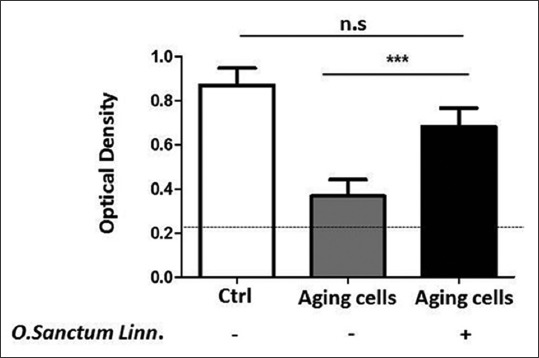
Choline acetyltransferase (ChAT) activity on the aging human cerebral microvascular endothelial cells with and without treatment of ethanolic extract *Ocimum sanctum* Linn. In house, ChAT sandwich enzyme-linked immunosorbent assay was performed to analysis the ChAT activity. Statistical analysis was performed by two-tailed Student’s t-test. All experiment were performed in duplicate (n=3. White column=Control positive ChAT activity; gray column=Aging untreated cells; black column=Aging treated cells).

To analysis the activity of ChAT on the aging brain cells after treatment, sandwich ELISA was performed, and from the ELISA absorbance results could be described that the ChAT activity on the aging cells after treatment in significantly increased (OD; 0.682) as same as young age model cells (OD; 0.866). In the other hand, nontreated aging cells showed very low ChAT activity (0.368) (Figure-4).

## Discussion

Memory, cognitive and/or learning ability is the most important part of human being sustainability. Recently, memory and cognitive loose know as dementia not only occur during aging but also frequently become serious clinical sign from ischemic brain damage, head injuries and also for neurodegenerative disease, e.g., Alzheimer disease, Parkinson disease [11]. Nowadays, medication and prevention for dementia are still far from satisfactory. Thus, it is needed to explore and investigate a new agent as a drug candidate for memory impairment. *O. sanctum* Linn. is potential herbs which are easily found and grow all around the world not only in Indonesia but also in tropic and subtropic country. *O. sanctum* Linn. containing several active substances including rosmarinic acid, ursolic acid, flavonoids and tannins, eugenol, luteolin, apigenin, b-caryophyllene, methyl eugenol, and b-pinene [13,22-24]. That’s why almost of the part of *O. sanctum* Linn. gives a lot of advantages as a medication [12,13,22,25,26]. However, currently only limited data already known regarding the curing effect of *O. sanctum* Linn. in neurodegenerative diseases [11,18]. In addition restricted finding describe the involvement of noncholinergic cells on the mechanism of learning and memory.

In this presents, the study in our *in vitro* model using HCMECs described that ChAT expressed on the intracytoplasmic area from the human cerebral endothelial cells (Figure-1). HCMECs is a cell line which is derived from human temporal lobe microvessels isolated from tissue excised during surgery [27-29], and the HCMECs are used widely as an *in vitro* model for blood–brain barrier [27]. Blood-brain barrier on the human being are tightly regulated interface with central nervous system especially with astrocytes and pericytes and form the neurovascular unit [30]. In addition, other researches already describe that enzyme ChAT is expressed not only on the neuronal cells but also non neuronal cells with barrier and immune functions [31,32]. Since ChAT enzyme is precusor of Ach, thus the expression of ChAT is in line with the expression of Ach. Meanwhile, the expression of Ach may help to maintain and to optimize cell function, such as proliferation, differentiation, formation of a physical barrier, migration, and ion and water movements. Blockade of n- and muscarinic acetylcholine receptors on noninnervated cells causes cellular dysfunction and/or cell death (apoptosis). Thus, cholinergic signaling in nonneuronal cells is comparable to cholinergic neurotransmission. Dysfunction of the nonneuronal cholinergic system may involve in the pathogenesis of diseases. Based on that evidence, we strongly believed in our experimental design on our models, ChAT which produced by endothelial cells (HCMECs) have a directly effect on the nervous system in here is on the brain tissue. Several numbers of research already described also that ChAT is localized in the rat cerebral microvascular endothelial cells and microglia [27-29,33,34]. Based on these finding, we can postulate that cerebral endothelial cell which is mimics of blood brain barrier may be is one of ChAT source in the brain derived from non-cholinergic cells. Our postulate is in line with several research which describes that there is a possibility of ChAT was produced from noncholinergic systems such as skin, colon, blood vessel (arteries), and human placenta [31,35-37]. Furthermore, the intriguing question, actually how is the role of ChAT which expressed on cerebral microvascular endothelial cells on the learning and memory mechanism? ChAT is transferase enzyme which is responsible for the synthesis of Ach. Ach is a neurotransmitter, which plays a significant role in memory and cognitive process. Mainly, ChAT is synthesized on the perikaryon of all cholinergic cells in the brain, and then transported by slow axoplasmic transport to the axon terminal, and available for the synthesis of Ach [8]. During Ach synthesis and release, both on the neuronal and endothelial sites are associated with microvessels in the cerebral cortex. Analog with the mechanism, in cerebral microvascular endothelial cells, choline were taken up by endothelial cells and converted to the presence of ChAT, and stores as Ach. In microvascular endothelial cells, Ach will be released gradually in the presence of nerve degeneration or damage, e.g., stress oxidative, hypoxia, stroke, intracerebral hemorrhage and destruction of the cholinergic neuron by amyloid β peptide [7]. The expression of Ach will give protection to the brain, nerve cells, and vessel wall. Thus, this neuroprotective function may help the stability live of the cells, avoid neurodegeneration and may enhance memory as well as cognitive ability [30-32,38]. Moreover, cerebral microvascular endothelial cells release substances which can promote neurogenesis in collaborated with astrocytes. This mechanism are pivotal to develop a niche which is needed to improve memory and cognitive ability on the neurodegenerative diseases [39,40].

**Supplemental Figure-1:**
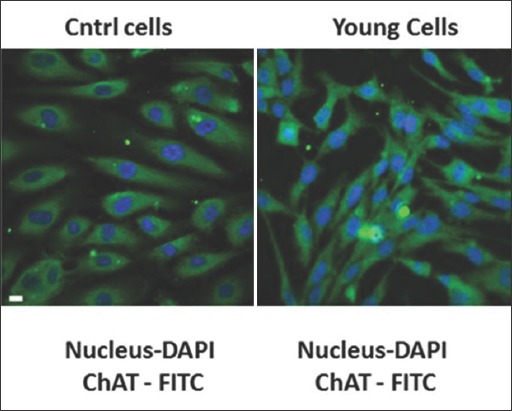
Choline acetyltransferase (ChAT) expression on the human cerebral microvascular endothelial cells (HCMECs) mimics young ages conditioned after and before treatment with *Ocimum sanctum* Linn. ethanolic extract. HCMECs were fixed and continued with incubation by using primer antibody goat anti ChAT (Clone AB144P). After overnight incubation, cells were washed and labeled by secondary antibody Alexa fluor fluorescein isothiocyanate (FITC) labeled donkey anti goat or Alexa fluor FITC labeled goat anti mouse, nucleus were stained with 4’,6-diamidino-2-phenylindole. (a) control cells before treatment, (b) treated cells. Blue=Nucleus; Green=ChAT.

In addition, in the treatment of ethanolic extract derived from the leaves of *O. sanctum* Linn. on the high passage HCMECs which is mimics aging conditions, we found that these extract capable stimulate and restore the expression of ChAT (Figures-2 and 3). However, the treatment of *O. sanctum* extract on the young ages cells may not give significantly increasing expressing of ChAT (Supplemental Figure-1 and Table-1), this evidence could give an explained that on the young ages the extract of *O. sanctum* only act to maintenance the stability of ChAT expression. Beside have functioned as the precursor of Ach, ChAT also presently as a specific marker for functional activity of cholinergic neurons both in central and peripheral nervous systems [9,41,42]. Some reports indicated cholinergic neuron are involved in neurophysics function such learning, memory, sleep, arousal, and movement. Meanwhile if there is occurs inhibition of the ChAT production and push down ChAT activity whether because of aging [35,36] or pathological condition such as oxidative stress, brain injury, stroke, degeneration cholinergic neuron by amyloid β peptide [7], its will directly disturb secretion of Ach and also induce decreasing amount of cholinergic neuron thus may impair memory, spatial learning and cognitive ability [37,43]. Moreover in our ELISA analysis (Figure-4) showed after administrated with *O. sanctum* ethanolic extract, the expression of ChAT on the aging cells is increasing, or maybe in here we can say the ethanolic extract induce restoring ChAT activity in the aging cells. Already known during aging ChAT activity will be decreased both on noncholinergic or cholinergic cells, but reducing the activity is not in line with cell degeneration. Usually, cells degeneration occurs because of pathological reasons such as Alzheimer, stroke, or hypoxia. Based on our finding, we can hypothetically mention that increasing the expression of ChAT is in line with the activity of this enzyme. Thus, we can propose in the proper condition of ChAT, and this enzyme may promote the production of Ach thus its may enhance and or restore memory and cognitive ability in aging individual.

**Supplemental Table-1:** The semiquantitative number of ChAT expressions.

Cell conditions	HCMECs	

	Young	Young and *O. sanctum* Linn. extract
ChAT expressions	+++	+++

+++=Numerous, -=Not detected, +=A few, ChAT=Choline acetyltransferase, HCMECs=Human cerebral microvascular endothelial cells, *O. sanctum*=*Ocimum sanctum*

Based on these results, it is crucial to keep the stability ChAT expression not only in cholinergic cells but also on the noncholinergic cell primarily cerebral microvascular endothelial cells. It will be interesting to explore more deeply the ChAT mechanism in cellular level especially on the noncholinergic cells and may give a new perspective of dementia therapeutic target. *O. sanctum* Linn. is potentially drug candidate for dementia, however, and need to be consideration our research is based on *in vitro* model. The determination of the appropriate dosage from this herb to be applied on the *in vivo* model thus can mimics more closely to the environment of the human body is a need to be done.

## Conclusion

Taking together ChAT is expressed on the HCMECs. An ethanolic extract derived from leaves of *O. sanctum* Linn. may stimulate and restore the expression of ChAT on the aging cells. *O. sanctum* Linn. could give nerve protection and revealed production of Ach thus may enhance the memory and cognitive ability.

## Authors’ Contributions

DLK and HW designed the experiments and study; DLK, HW, and AH performed the experiments; DLK and HW interpreted the data and wrote the manuscript. All authors read and approved the final manuscript.
